# The Impact of Vibration Therapy Interventions on Skin Condition and Skin Temperature Changes in Young Women with Lipodystrophy: A Pilot Study

**DOI:** 10.1155/2019/8436325

**Published:** 2019-05-29

**Authors:** Wanda Pilch, Olga Czerwińska-Ledwig, Joanna Chitryniewicz-Rostek, Magdalena Nastałek, Piotr Krężałek, Dominika Jędrychowska, Natalia Totko-Borkusewicz, Ivan Uher, Dávid Kaško, Łukasz Tota, Anna Tyka, Aleksander Tyka, Anna Piotrowska

**Affiliations:** ^1^Faculty of Rehabilitation, University of Physical Education in Krakow, Poland; ^2^Science Club by Department of Biochemistry and Basics of Cosmetology, University of Physical Education in Krakow, Poland; ^3^Institute for Society and Health, Pavol Jozef Šafárik University, Košice, Slovakia; ^4^Institute of Physical Education and Sports, Pavol Jozef Šafárik University, Košice, Slovakia; ^5^Faculty of Physical Education and Sport, University of Physical Education in Krakow, Poland; ^6^Faculty of Tourism and Leisure, University of Physical Education in Krakow, Poland

## Abstract

**Background:**

Cellulite affects 85-98% of women aged over 20 years. In a given context, mechanical vibrations have not been applied in the therapy of gynoid lipodystrophy (GL) until now. The aim of this pilot study was to assess the condition and temperature of skin affected by cellulite after vibration therapy interventions in young women with GL.

**Methods:**

10 healthy women (21.5 ± 1.5 years old) with stage 1 or 2 Nürnberger-Müller scale of severity of GL participated in the study. The subjects underwent 20 vibration interventions with the use of a Rehabilitation Massage Device Vitberg+. Vibration therapy was applied 5 times a week for 60 minutes during 4-week period. Before and after first and last interventions, grade of lipodystrophy was assessed and thermographic images were taken.

**Results:**

Visual and palpation analysis performed before and after a series of treatments proved a total cellulite remission after the intervention among 40% of subjects (from stage 1 to stage 0). Among the remaining 60% with stage 2 of cellulite, an improvement in the skin condition was observed, and cellulite grade was determined as stage 1. The mean skin temperature in the lateral part of thigh as well as on the posterior surface of thigh and buttocks increased significantly (p<0.00001) after both the first (respectively: 4.0°C ± 0.9°C, 3.9°C ± 0.8°C) and the last vibration therapy interventions (respectively: 3.1°C ± 1.1°C, 2.8°C ± 1.1°C). After the series of interventions, a statistical significant (p=0.00705) increase in the mean skin temperature in the lateral thigh was observed—before the first treatment: 27.9°C ± 0.7°C; before twentieth treatment: 29.0°C ± 1.2°C.

**Conclusion:**

The series of vibration interventions contributed to the reduction of GL among the participants. The thermographic imaging analysis proved an impact of both single and serial vibration interventions.

## 1. Introduction

Cellulite (gynoid lipodystrophy, GL) is a fibromatous degeneration of the connective tissue [1, 2]. GL affects 85-98% of women of all ethnicities after the onset of puberty, which suggests a hormonal component of its pathogeny. It can occur on the surface of any part of the body that contains subcutaneous adipose tissue. The superior and posterior regions of the thighs and buttocks are the most susceptible areas. It usually begins with microcirculation disorders and dysfunction of blood and lymphatic vessels with increase in their permeability. This leads to disturbances in the supply of nutrient to the cells. Furthermore, it is a factor in lymphatic drainage disorder and excessive collection of fluids in interstitial sites [3]. The main theories that aim to explain GL pathophysiology embrace a modification in the connective tissue septa, the arrangement of the skin, and vascular changes. Depending on the severity of the appearance, the condition may cause significant psychosocial disturbance. A number of therapies have been clinically tested and many have been withdrawn, not solely for their therapeutic ineffectiveness, but also for their risk to subjects' health. Some treatments have weak scientific basis. However, some are supported by actual scientific evidence, e.g., radiofrequency, intense pulse light, ultrasound, shockwave therapy, endermology method, and carboxytherapy [2].

The effectiveness of intervention to advanced stages of GL is quite limited; generally, lowering of the GL signs is only possible [2, 4]. Therefore, the crucial factor is prophylaxis which incorporates an appropriately balanced diet, proper care, and physical activity [3]. Regularity is of primary importance as it leads to long-lasting effects in the form of lowering adipose tissue mass, increasing muscle mass, and improving skin tension [5].

Beauty parlors offer numerous anticellulite interventions. Their diversity concerns the instruments and materials used, but they involve three common directions of action: microcirculation improvement, enhancing lipolysis, and restoration of skin and blood vessel walls [2, 4, 6]. So far, local mechanical vibrations have not been used in GL therapy.

Vibration therapy was a method known back in ancient times. Today, many devices that apply vibrations are accessible, most often with fixed parameters describing the generated vibrations. The impact of vibration on the human body is multidirectional. Among others, vasodilatation is observed, resulting in an improvement in blood and lymph circulation [7], as well as a change in muscle tension through reflex activation [8]. It is also confirmed that vibration therapy exerts an antalgic effect and leads to an increase in muscle mass and bone density [9–11]. One of the results of vibration massage is a rise of temperature within the skin and subcutaneous tissues in the regions covered with the intervention [12]. Many of the enumerated mechanisms can turn out efficient in reducing signs of GL.

Among the referenced methods in body temperature studies is thermography, a contactless technique of infrared radiation registration. Medium-infrared tissue emissivity and homeothermy makes the human body a convenient object for thermographic research. Visualization of temperature distribution can be valuable diagnostic information, and thus thermography is now widely applied in various fields of medicine [13–15].

The aim of presented study was to assess the skin condition and changes in skin temperature in body areas affected by cellulite after series of vibrotherapy treatments. The applied method was thermographic imaging after single and serial vibration therapy interventions among young women with grade 1 or 2 (stage 1: not evident in the standing or lying positions; however, pinch test results in a dimpled appearance; stage 2: spontaneously apparent in the standing position, but not in the lying position [1]) lipodystrophy on the Nürnberger-Müller scale.

## 2. Materials and Methods

### 2.1. Study Group Characteristics

The pooled data (n=10) were extracted from the women students of the University of Physical Education in Krakow, Poland, aged 21–23 years (mean age of all participants was 21.5 ± 1.5).

The study was conducted in 2018. Due to a number of factors affecting the severity of cellulite symptoms, the criteria for inclusion in the study are listed in Table 1. Qualification according to the above criteria ensured homogeneity of the studied group. The main inclusion criterion comprised the diagnosis of stage 1 or 2 GL on the Nürnberger-Müller scale [1]. Physical activity, being an inclusion criterion, was assessed with the International Physical Activity Questionnaire (IPAQ) (short form) [16]. Physical Activity Level (PAL) was calculated as a ratio of total and resting energy expenditure within 24 hours, in reference to the part of total energy expenditure resulting from physical activity [17]. Included were subjects characterized by a low PAL (<1.6) and appropriate diet. Another inclusion criterion was a diet that did not differ significantly from the norms shown by Jarosz et al. [17]. A 5-day nutrition analysis was performed based on the latest guidelines for the Polish population [17]. The energy value of the diets composed by the participants equalled 1966.98 ± 556.32 kcal, while the amount recommended by the National Food and Nutrition Institute for the PAL of 1.6 equals 2100 kcal. In 7 subjects, the diet energy value was lower than that recommended. The studied subjects declared the dietary intake of 0.75 ± 0.13 g of proteins per kilogram body weight. The dietary analysis pointed at the daily intake of 78.65 ± 22.17 g of fat and 243.84 ± 46.62 g of carbohydrates, respectively. The values of the analyzed nutrition parameters (energy, proteins) were slightly lower, and the amount of dietary fat and carbohydrates turned out slightly higher than the recommended values.

Body composition parameters were estimated by the use of electrical bioimpedance (Tanita, Korea) and body height with an anthropometer (Martin, USA). The following measurements were recorded for each participant: body mass (BM), fat mass (FM), lean body mass (LBM), and total body water (TBW). The BMI coefficient was calculated. Women with a similar body composition were qualified for the project (Table 2).

The exclusion criteria are presented in Table 1, and contraindications to vibration intervention include pregnancy, metastatic malignancies, cardiac pacemaker, acute inflammatory conditions, cardiovascular diseases, thrombosis, convalescence period after hip or knee joint endoprosthesis or other musculoskeletal system interventions, acute back pain, advanced diabetes, and infectious diseases.

### 2.2. Study Methods

On the base of a visual and palpation examination performed by a dermatologist, the severity of lipodystrophic changes present in the thigh and buttock areas was assessed by means of Nürnberger-Müller scale [1] before and after the vibration therapy interventions.

In each subject, a series of thermographic images were obtained with a Thermo GEAR G120EX (NEC Avio Infrared Technologies Co., Ltd., Tokyo, Japan; detector: microbolometer, resolution 320x240 pixels, sensitivity 0,04 (at 30°C), accuracy ±2°C). The images were acquired after a 30-minute acclimatization to the conditions existing in the room before the first and the last intervention, as well as 10 minutes after the first and the last intervention, with fixed room temperature (22°C), low humidity (45-50%), and low fixed lighting in stable distance. Images of each participant's lateral part of the right thigh and the posterior surface of thighs and buttocks were taken. The distance between the subject and the camera was 120 cm. The thermograms were analyzed by calculating the mean temperature in the studied body areas with the use of software for colorimetric assessment of images, InfReC Analyzer NS9500 Standard (NEC Avio Infrared Technologies Co., Ltd., Tokyo, Japan). The images and thermograms analysis were obtained in accordance with the recommendations of the European Association of Thermology.

### 2.3. Vibration Therapy Intervention

**Figure 1:**
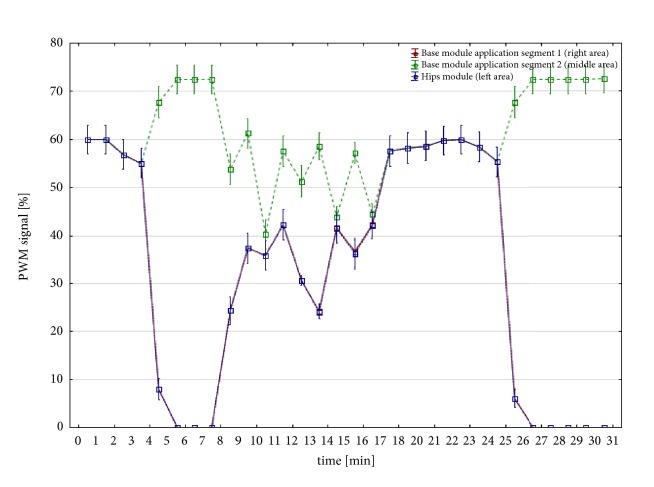
Voltage-current characteristics of the Hips program with the Hips module (PWM: Pulse Width Modulation).

**Figure 2:**
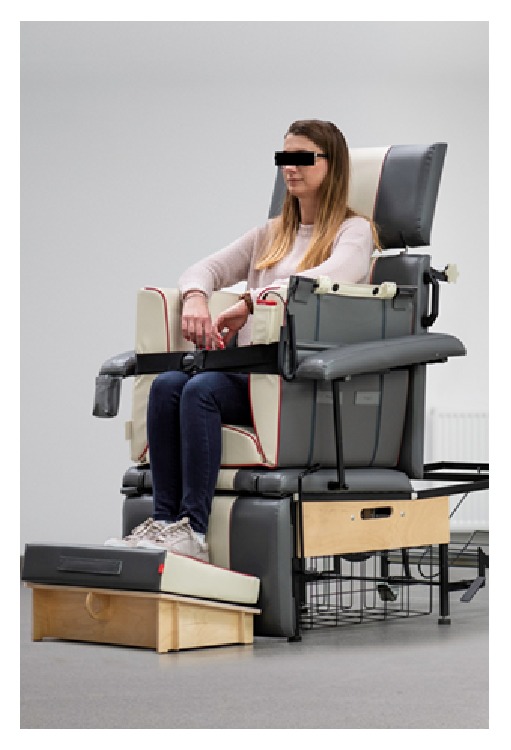
Position of the subject during vibrotherapy treatment.

The series of vibration therapy interventions were applied within 4 weeks, Monday through Friday, once a day for 60 minutes (duration of treatment was determined with the accordance to manufacturers' information based on unpublished marketing tests). Each treatment was conducted maintaining the same protocol. It consisted of 2 cycles with variable parameters (Table 3).  Figure 1 shows detailed characteristics of a single cycle—pulse width modulation (PWM) against time. The source of the vibration stimulus was a Rehabilitation Massage Device Vitberg+ (Poland) (Basic module + Hips module, class II a medical device), with grade IV (maximal) intensity as defined by the manufacturer. Said device is certified by the notified body TUV Rheinland (no. 0197). The product also has a quality certificate for medical devices (no. HD 601181190001). Additionally, it is the only product available in Europe that enables to perform treatment in a sitting position, which allows to apply vibration stimulus both in a local and in a systemic manner. The intervention was performed in the sitting position, and subjects were wearing underwear which allowed direct contact between the surface of the massage device and the skin (Figure 2). The position of the body was controlled by a physiotherapist.

### 2.4. Statistical Analysis

All the analysis was performed with the Statistical 10.0 software (StatSoft, USA). The results are presented as means ± standard deviations. The Shapiro-Wilk test served to assess the normality of distribution. The differences between the results achieved before and after the therapy for standard distribution variables were analysed with Student's* t*-test for dependent groups; for variables with other types of distribution, the Wilcoxon test for paired samples was applied. Statistical significance was set at (*P* < 0.05).

### 2.5. Ethics Statement

The study was approved by the Ethical Committee of the Regional Medical Chamber in Krakow, Poland (approval No. 36/KBL/OIL/2018). The project was registered in Australian New Zealand Clinical Trials Registry (ANZCTR), number 12619000092190. Informed consent was submitted by all subjects when enrolled.

## 3. Results

The body composition parameters of the participants were correct and fit within the average values for women of the given age (Table 2).

**Figure 3:**
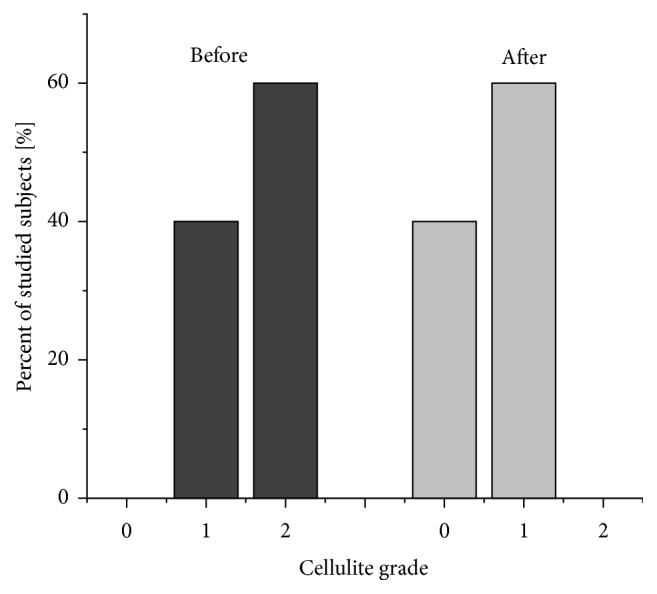
The changes of the cellulite grade (Nürnberger-Müller scale) in studied subjects before and after four-week vibration therapy interventions.

In the group included in the study, 6 participants (60%) had grade 2 cellulite (skin unevenness visible only in the standing position; skin smooth in the prone position) and 4 (40%) had grade 1 cellulite (skin smooth in both the standing and prone position; unevenness visible after skin compression only). Fat-related (6 subjects), water retention (1 subject), and mixed (3 subjects) cellulite were observed. The visual and palpation analysis after the vibration therapy completion showed a total cellulite remission among 40% of subjects with stage 1 GL. Among the remaining 60%, an improvement in the skin condition was observed, and GL was qualified as grade 1 on the Nürnberger-Müller scale.  Figure 3 presents observed changes. Statistical data is shown in Table 4.

**Figure 4:**
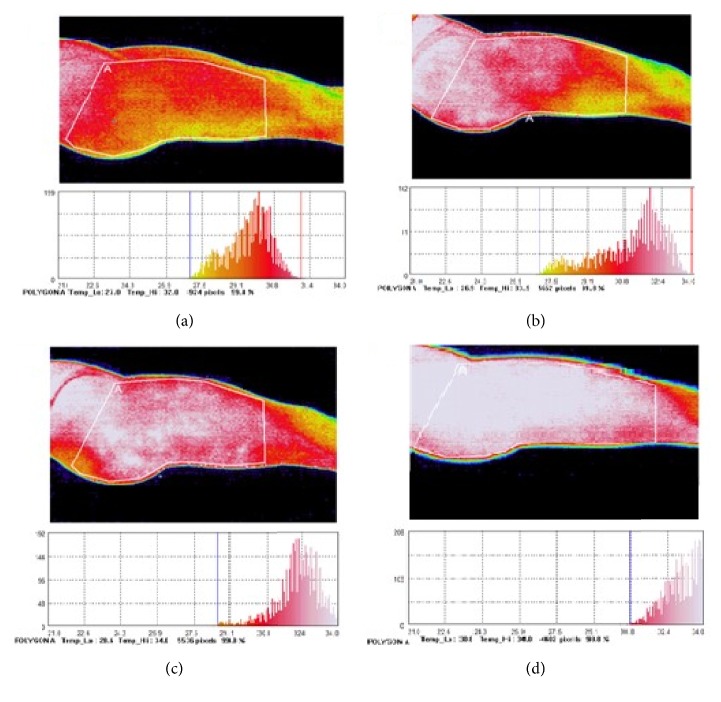
Thermograms of the lateral part of the thigh of one of the subjects before the beginning of the first vibration treatment (a), 10 minutes after its completion (b), before the beginning of the 20th session (c), and 10 minutes after (d).

Based on the thermographic images obtained before and after the first and twentieth intervention, the mean skin temperature was calculated for the lateral part of thigh, as well as for the posterior surface of thigh and buttocks. Exemplary thermographic images are presented in Figure 4.

**Figure 5:**
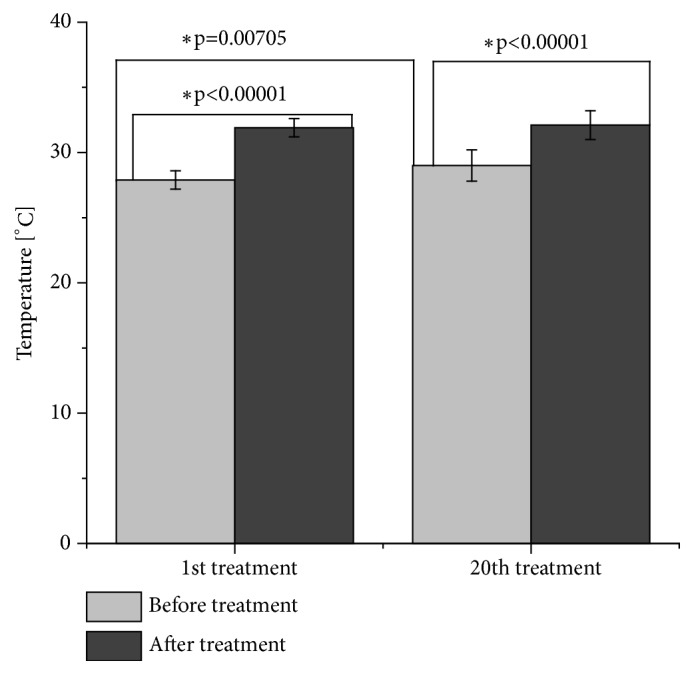
Mean changes of the temperature of lateral parts of the thighs during a series of vibration therapy treatments (*∗*p<0.05, t-test for dependent groups).

The mean temperature of the lateral part of thigh among the 10 participants before the first intervention was 27.9°C ± 0.7°C. After the first intervention, it increased significantly by the mean of 4.0°C ± 0.9°C (p<0.00001) and equaled 31.9°C ± 0.7°C. The highest temperature increase was observed in the third subject (5.6°C). Before the twentieth intervention, the mean temperature of the lateral part of thigh reached 29.0°C ± 1.2°C. After the intervention, it increased significantly by the mean of 3.1°C ± 1.1°C (p<0.00001) and equaled 32.1°C ± 1.1°C. The highest temperature increase, by 4.9°C, was observed in the seventh subject. The analysis of the mean temperature before the first and the twentieth intervention showed a statistically significant increase by 1.0°C (p=0.00705). Substantial difference occurred in the eighth participant (3.5°C). The results are depicted in Figure 5.

**Figure 6:**
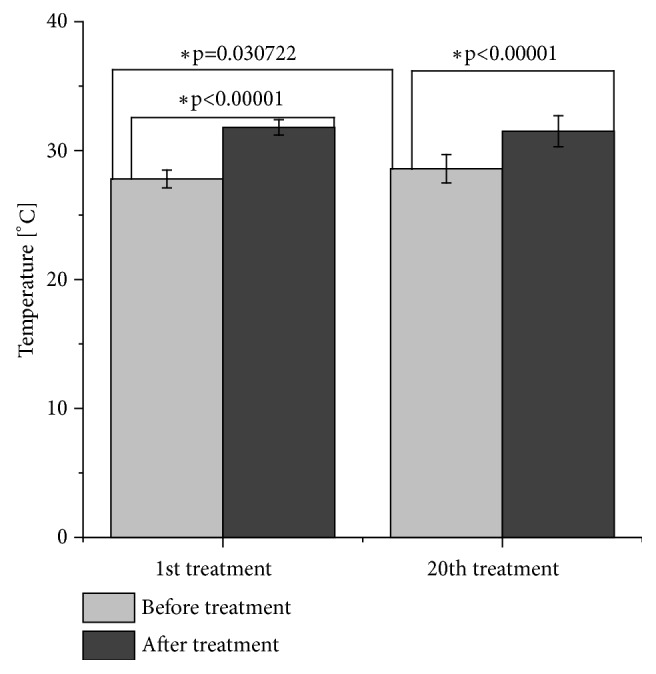
Mean changes of the temperature of back of the thighs and buttocks during a series of vibration therapy treatments (*∗*p<0.05, t-test for dependent groups).

After the first intervention, the mean temperature of the posterior part of thigh and buttocks increased significantly by 3.9°C ± 0.8°C (p<0.00001). In the fourth subject, the biggest temperature increase was observed (by 4.8°C). After the twentieth intervention, the mean temperature turned out significantly higher by 2.8°C ± 1.1°C (p<0.00001). The highest increase (by 4.2°C) occurred in the seventh and ninth participants. The mean temperature before the twentieth intervention was 0.7°C ± 1.1°C higher than that observed before the first intervention, and the increase was statistically significant (p=0.030722). The highest increase (by 2°C) was found in the second study subject. The results are presented in Figure 6.

The analysis of thermographic images proved that, both after the first and after the last intervention, the skin temperature in the areas affected by GL was higher than before the interventions. In 35% of the study participants, the difference in the mean temperature before and after the vibration therapy intervention did not exceed 3°C. In 35% of cases, the difference equaled 4°C or more. In the remaining group of women, the mean temperature difference ranged from 3°C to 4°C. Among 80% of the subjects, the skin temperature in the regions affected by cellulite increased between the first and last intervention.

## 4. Discussion

Presently, therapeutic vibration is utilized with the use of numerous purposes. However, the available researches refer mainly to the whole body vibration (WBV) interventions. With the stimulus applied mostly in the standing position on a vibrating platform, the local efficiency is limited. Also, a considerable part of the vibration stimulus is absorbed by the skeletal system. One can presume that vibration therapy activates the mechanism of adipose tissue burning [18]. It has been proven that WBV can be a tool to obtain a change in the body composition in obese women, as well as to improve muscle strength, with maintained bone density [11, 19]. Moreover, the combination of calorie restriction and long-lasting WBV training has turned out equally effective as the combination of diet and aerobic exercise to improve body composition in obese women [20]. GL changes are mainly characterized by considerable microcirculation disorders. Furthermore, insufficient tissue vascularization can result in its weak nutrition and oxygenation. Moreover, cellulite affected skin can become dry and cold [4]. Vibration therapy can be associated with a reduction of adipose tissue and blood flow enhancement.

The heat phenomena on the skin surface plane result from the metabolic changes within the tissues lying under its surface, the level of blood supply in these tissues, the thermal conductivity of the muscle and adipose tissues, and the heat transfer through the skin to the environment, including gas exchange [21]. In the presented study, thermographic imaging was applied to assess the effect of vibration on tissues and determine the resulting skin temperature changes. In our research, we observed the reduction in grade of cellulite measured in Nürnberger-Müller scale and an increase in skin temperature after the 4-week treatment period. As it was shown in previously published papers [22, 23], contactless thermography can be widely applied to diagnose cellulite and other skin disorders involving surface temperature changes. Utility of thermographic imaging as a tool for monitoring of effectiveness of cellulite therapy with use of cosmetic products was assessed in work of Migasiewicz et al. [22]. Local skin temperature depends considerably on the muscle and adipose tissue thickness, which significantly influences heat transmission throughout skin from the tissues lying deeper to the environment [24]. The mean temperature at the beginning of the vibration therapy equaled 27.9°C in the lateral part of thigh and 27.8°C in the posterior part of thigh and buttocks; both results were lower than those obtained by Zaproudina et al. [21] for the same skin areas in young men. The mean temperature measured in our study 10 minutes after the end of a session was higher than observed by Sonza et al. [25], where the impact of the vibration platform on the skin temperature of the back of the thigh was studied. In this study, 4 different vibration frequencies were evaluated, and the temperature of the posterior of the thigh was between 28 and 29°C, while in our subjects the mean thigh temperature was 31.8°C. These alterations partially resulted from the differences in the measurement protocol and differences regarding gender and the amount of adipose tissue with thermal isolation properties in the studied body regions of the subjects. The one-off vibration massage intervention significantly influenced the increase of skin temperature through vibration-induced mechanical friction, as well as muscle cells contractions (tonic vibration reflex), intensifying metabolic changes, which are accompanied by temperature increase [26]. We suppose that the increased skin temperature observed in our study is a mutual effect of the muscles contractions stimulated by tonic vibration reflex and the friction generated by vibrations applied locally. Both processes lead to the expansion of the vascular bed, which was observed as an increase in skin temperature. The significance of this phenomenon is underlined by the fact that, among ingredients used in cosmetics dedicated to skin with cellulite, there are popular ingredients that improve blood flow in the affected tissues [27, 28].

The series of 20 vibration interventions resulted in an increase in the mean temperature of the lateral part of the thigh which suggests long-term effect of vibration applied locally. The repeated use of vibrations applied on the legs and buttocks can increase the mean temperature that influenced adipose tissue in these areas. As a result, adipose tissue becomes thinner or gains better blood supply [29]. However, experimental observations concerning that effect are limited and further studies are needed to support this hypothesis.

In already published papers on reducing the severity of cellulite symptoms, only systemic vibration (WBV) has been applied. These studies indicate the beneficial effect of this type of stimulus, assessed thermovision and subjective assessment of participants [24]. The increase in skin temperature can, therefore, allow indirect conclusions on the improvement of skin nutrition and vascularization. The month-long vibration intervention caused a significant increase of the lateral thigh temperature, which may point to the increase of blood supply to this area and, therefore, further intensify metabolic changes within the adipose tissue. The effectiveness of this method of vibration application in the augmentation of therapeutic effects of other forms of cellulite therapy, for example, electrical stimulation combined with ultrasound [30] or low-level laser therapy [31], has also been indicated. Nevertheless, vibration applied locally used in our study has less contradictions than WBV platforms, which are burdened with higher risk of side effects. The vibration stimulus used locally could have stronger effects because it is not absorbed by skeletal system.

Nakagami et al. [7] observed in their study that intervention with the use of vibrations improved skin blood supply in young subjects, without direct impairment of the musculoskeletal or circulatory systems. Cyclic tensions and loading exerted on the endothelial surface of an artery during vibration therapy can stimulate the release of beneficial mediators such as nitric oxide (NO), which can cause vasodilatation [32]. Moreover, improved muscle condition, which constitutes an advantage of vibration therapy, is a significant factor supporting fight with fat-related cellulite. By increasing skin microcirculation, the vibration intervention with the Hips module of the massage device applied in the study can improve skin oxygenation and tone, as well as support lipodystrophy reduction, also through an improvement of lymph circulation and the micromassage, generated in the tissue during an intervention [31, 32]. Vibration massage is plausible, among others, for cohorts with the problem of skin dryness [33]. In the course of our study, the participants repeatedly reported an improvement in the general skin condition—that is moistness, elasticity, and softness—which indicates the need for additional investigation in this area.

The results of the presented study seem to prove that vibration therapy helps to reduce GL, mainly through decreasing microcirculation disorders, underlying the development of cellulite. One should not exclude, though, the impact of other mechanisms, such as lymphatic drainage, influence on the adipose tissue metabolism in the thigh and buttock regions, or improvement in the operation of muscles and fascial tissue in the area affected by GL. Additionally, our previous work shows that oscillating-cycloidal vibrations applied locally and systemically can affect lipid metabolism [34] and improve insulin sensitivity [35]. Both of these mechanisms seem to be useful in reducing cellulite symptoms. Further studies, however, are needed in this field.

In conclusion, we can express an opinion that the series of vibration interventions contributed to the reduction of GL in the selected sample. The thermographic imaging analysis proved an impact of both single and serial vibration interventions on the skin temperature in body areas affected by cellulite, which can be associated with improved blood flow within the skin, and a further supply of nutrients to the said area of the human body. In our opinion, especially the long-term effect observed in our study seems to be important in cellulite therapy. Therefore, vibration therapy with a local application can to a certain extent play a significant role in diminishing GL. However, further studies are needed to clarify a complex interplay among vibrotherapy, GL, and skin temperature.

## Figures and Tables

**Table 1:** Inclusion and exclusion criteria.

Inclusion criteria	Exclusion criteria
Stage 1 or 2 of cellulite on the Nürnberger-Müller scale	Lack of cellulite lesions or lesions in stage 3 on the Nürnberger-Müller scale

Low physical activity level (PAL<1.6)	History of endocrine disorders

Age between 21 and 23 years	Special or elimination diets applied up to 3 months preceding the project

Female sex	Contraindications to vibration intervention

**Table 2:** Anthropometric features of the studied women (n=10).

Characteristics	Values
Body mass [kg]	62.5 ± 4.5

Fat mass [kg]	17.0 ± 3.5

Lean Body Mass [kg]	45.1 ± 2.7

Total Body Water [%]	33.1 ± 2.35

BMI [kg/m^2^]	22.8 ± 1.1

Values are presented as means ± SD.

**Table 3:** Parameters of the Rehabilitation Massage Device RAM Vitberg+ in the Hips program (empirical results provided by the manufacturer).

Ranges (min-max)		
a[m/s^2^]	A[mm]	f[Hz]
1.1 - 27	0.01 - 0.58	17.5 - 46.5

a: acceleration, A: amplitude, and f: frequency.

**Table 4:** Changes in cellulite grade (Nürnberger-Müller scale) in studied subjects (*∗*p<0.05, Wilcoxon test).

	Before	After
Mean:	1.6±0.5	0.6±0.5
	*∗*p=0.00256	

Values are presented as means ± SD.

## Data Availability

The data used to support the findings of this study are available from the corresponding author upon request.
